# CXCR2 deficiency impairs auditory function in spiral ganglion neurons by altering excitability

**DOI:** 10.1038/s41420-026-03366-7

**Published:** 2026-10-15

**Authors:** Fuyu Xian, Xueya Ma, Zhongyang Zhang, Cailin He, Zeyuan Li, Jiaxi Liu, Xishuo Jin, Jundan Xiao, Haitao Shen, Ping Lv

**Affiliations:** 1 Department of Pharmacology, The Key Laboratory of Neural and Vascular Biology, Ministry of Education, The Key Laboratory of New Drug Pharmacology and Toxicology, The Hebei Collaboration Innovation Center for Mechanism, Diagnosis and Treatment of Neurological and Psychiatric Disease, Hebei Medical University, Shijiazhuang, China; 2 Lab of Pathology, Hebei Medical University, Shijiazhuang, China

**Keywords:** Auditory system, Ion channels in the nervous system

## Abstract

CXC chemokine receptor 2 (CXCR2), a key member of the chemokine receptor family, plays a central role in regulating immune and inflammatory responses. Inflammation has been established as a critical pathological factor in various auditory disorders, including noise-induced hearing loss, drug-induced ototoxicity, and age-related hearing loss; however, the expression patterns and biological functions of CXCR2 in key cells involved in auditory signal transduction remain unclear. Here, we demonstrated that CXCR2 is abundantly expressed in cochlear spiral ganglion neurons (SGNs), hair cells, and the stria vascularis. Genetic deletion of CXCR2 resulted in progressive hearing loss in mice. SGNs from CXCR2 knockout mice exhibited increased expression of the Kv1.2 potassium channel, impaired ribbon synapses, and altered myelin integrity. Electrophysiological analysis revealed enhanced Kv1 currents and significantly reduced excitability in CXCR2-deficient SGNs. Notably, CXCR2 knockdown in SGNs using an adeno-associated virus recapitulated the hearing loss phenotype. In conclusion, our findings revealed that CXCR2 is essential for hearing as it regulates SGN excitability.

## Introduction

Hearing loss is one of the most common sensory disabilities worldwide, affecting over 6.8% of the world’s population [1]. Cochlear cell damage, induced by noise, aging, and ototoxicity, is a major contributor to deafness. Spiral ganglion neurons (SGNs) are responsible for transmitting complex acoustic information to the central auditory system to process sound. Their structural and functional integrity constitute the core biological foundation of cochlear implantation. However, mammalian SGNs lack regenerative capacity, and their degeneration leads to permanent hearing impairment [2]. Consequently, understanding the regulatory mechanisms underlying SGN function is essential for developing therapeutic strategies for hearing loss.

Recent studies have revealed that macrophages promote the long-term survival of SGNs and repair damaged ribbon synapses via neuron-immune fractalkine (CX3CL1–CX3CR1) signaling [3, 4]. Additionally, the chemerin/CMKLR1 pathway regulates apoptosis and inflammatory responses in cisplatin-induced SGN injury through the NF-κB signaling pathway [5]. These findings suggest that the chemokine system is critically involved in the modulation of SGN function. Notably, chemokines display dual roles in maintaining physiological homeostasis and driving pathological inflammation [6, 7]. Their receptors are highly expressed in non-immune cells [8] in addition to immune cells, implicating pleiotropic functions beyond immune regulation. Therefore, elucidating the roles of these receptors in SGNs is critical for understanding the chemokine systems and developing targeted immunotherapies.

The CXC chemokine receptor 2 (CXCR2) belongs to the CXCR subfamily. It was originally found in immune cells, such as neutrophils [9], monocytes [10], and natural killer cells [11], which are traditionally associated with inflammatory responses. However, emerging evidence has revealed its widespread expression in neurons [8], fibroblasts [12], and smooth muscle cells [13], indicating its diverse pathophysiological roles. Functionally, CXCR2 activation promotes angiogenesis and consequent tumor growth [14, 15], while its blockade attenuates CXCL1-induced heat hyperalgesia [16]. CXCR2 is a key molecule in the pathway downstream of p53 mediating cellular senescence [17]. A recent study indicated that CXCR2 knockdown in macrophages protected against cisplatin ototoxicity, indicating its potential involvement in hearing [18]. However, whether CXCR2 is expressed in cochlear SGNs and whether it contributes to auditory signal regulation currently remains unclear.

In the present study, we demonstrated that CXCR2 is highly expressed in cochlear SGNs, hair cells (HCs), and the stria vascularis of the cochlea. The genetic ablation of CXCR2 results in reduced SGN excitability and impaired hearing. Mechanistically, we found that CXCR2 deficiency led to upregulation of the Kv1.2 potassium channel, while pharmacological inhibition of the Kv1 channel increased SGNs' excitability. CXCR2 knockdown, specifically in SGNs, recapitulated hearing loss, mirroring the phenotype observed in global knockout mice. Collectively, these results establish a critical role for CXCR2 in auditory function, in which it maintains normal hearing by modulating SGN excitability through the Kv1.2 channel.

**Fig. 1:**
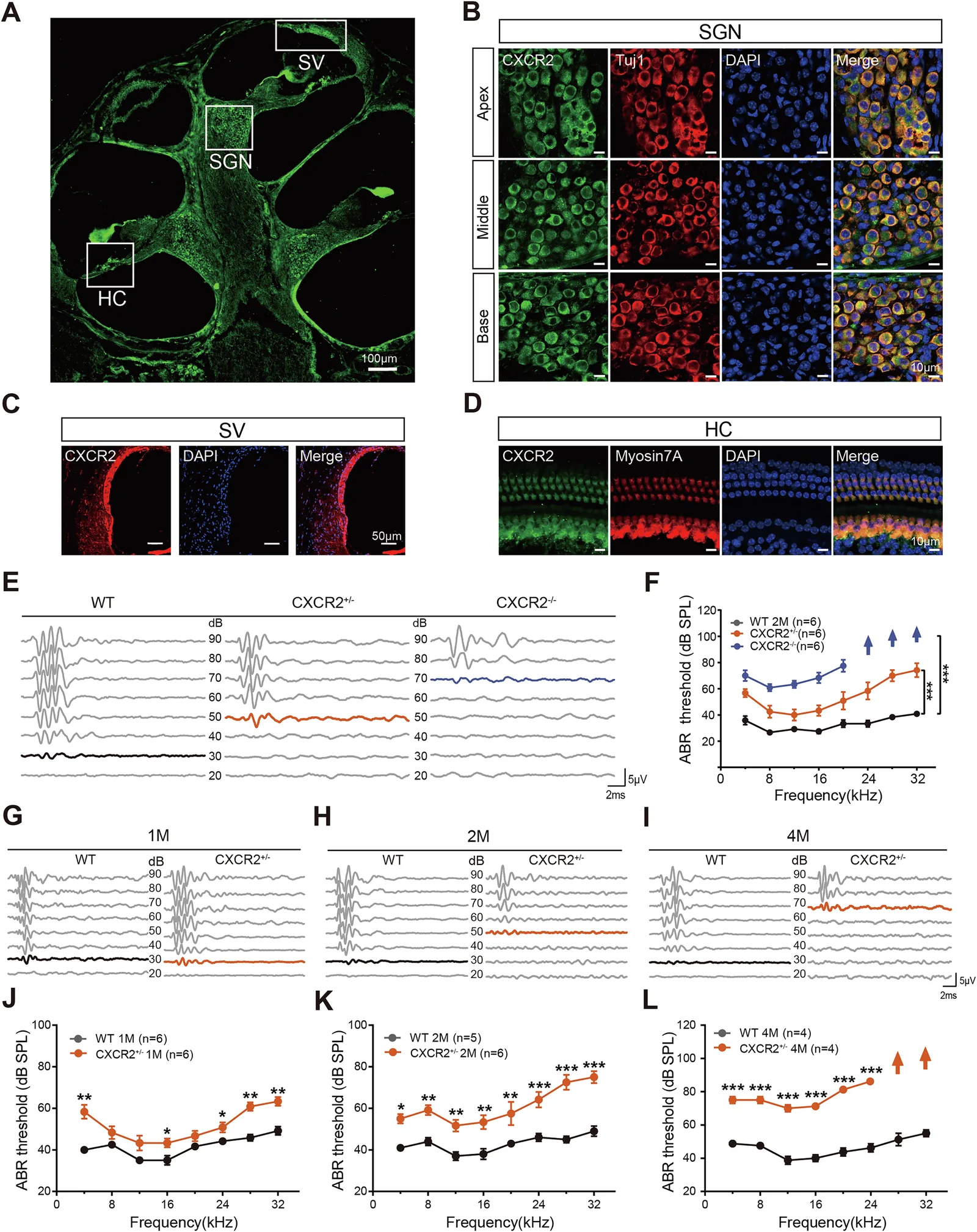
CXCR2 expression in the cochlea is essential for hearing. **A** Immunofluorescence staining of CXCR2 (green) in cross sections of the whole cochlea of 2-month-old WT mice. Scale bar: 100 μm. **B** Expression of CXCR2 in spiral ganglion neurons (SGNs) from the apical, middle, and basal cochlea of 2-month-old WT mice. SGNs were stained using anti-CXCR2 (green) and anti-Tuj1, a neuron marker (red). The nuclei were stained with DAPI (blue). Scale bar: 10 μm. **C** and **D** Expression of CXCR2 in stria vascularis (SV) and hair cells (HCs) in the cochlea of 2-month-old WT mice. Scale bar: 50 μm in (**C**), 10 μm in (**D**). **E** Representative ABR waveforms in response to click stimuli in 2-month-old WT, CXCR2^+/−^, and CXCR2^−/−^ mice. **F** ABR threshold statistics of WT, CXCR2^+/−^, and CXCR2^−/−^ mice at 2-month-old in response to pure tone stimuli. **G–I** Representative ABR waveforms respond to click stimuli in 1-, 2-, and 4-month-old WT and CXCR2^+/−^ mice. **J–L** ABR thresholds were measured in WT and CXCR2^+/−^ mice at 1-, 2-, and 4- months old. Data are means ± SEM, *p < 0.05, **p < 0.01, ***p < 0.001. Statistical analyses were performed using two-way ANOVA (**F**, **J**–**L**) with a Bonferroni post hoc test.

## Results

### CXCR2 is expressed in the mouse cochlea and is essential for hearing

To investigate the spatial distribution of CXCR2 in the auditory system, we performed whole-mount immunostaining of the cochlear tissues of 2-month-old wild-type (WT) mice. Our results revealed strong CXCR2 expression in cochlear SGNs, HCs, and the stria vascularis (Fig. 1A–D). Tuj1 and Myosin 7A were used as markers to stain for SGNs and HCs, respectively (Fig. 1B, D). Additionally, temporal expression profiling revealed that CXCR2 exhibited relatively stable expression levels in SGNs during development (Fig. S1A-B). To explore the role of CXCR2 in hearing, we generated CXCR2 constitutive knockout mice (Fig. S2), and auditory function was evaluated by measuring the auditory brainstem responses (ABR). Compared with WT mice, both heterozygous (CXCR2^+/-^) and homozygous CXCR2 knockout (CXCR2^-/-^) mice at 2 months of age exhibited a significant increase in the ABR threshold, indicating hearing impairment (Fig. 1E, F). Because of the high lethality in CXCR2^-/-^ mice, we performed subsequent experiments using CXCR2^+/-^ mice.

To examine whether the regulation of auditory function by CXCR2 is age-related, we evaluated the hearing function of CXCR2^+/-^ mice at 1-, 2-, and 4-months of age. Compared with age-matched WT mice, CXCR2^+/-^ mice displayed elevated ABR thresholds at 1 month of age, which further increased at 2 and 4 months, indicating progressive hearing loss (Fig. 1G–L). Furthermore, there were no detectable ABRs at 28 kHz and 32 kHz in 4-month-old CXCR2^+/-^ mice, indicating complete deafness in the high-frequency range (Fig. 1L).

### CXCR2^+/−^ mice at 2 months of age show no cochlear degeneration

**Fig. 2:**
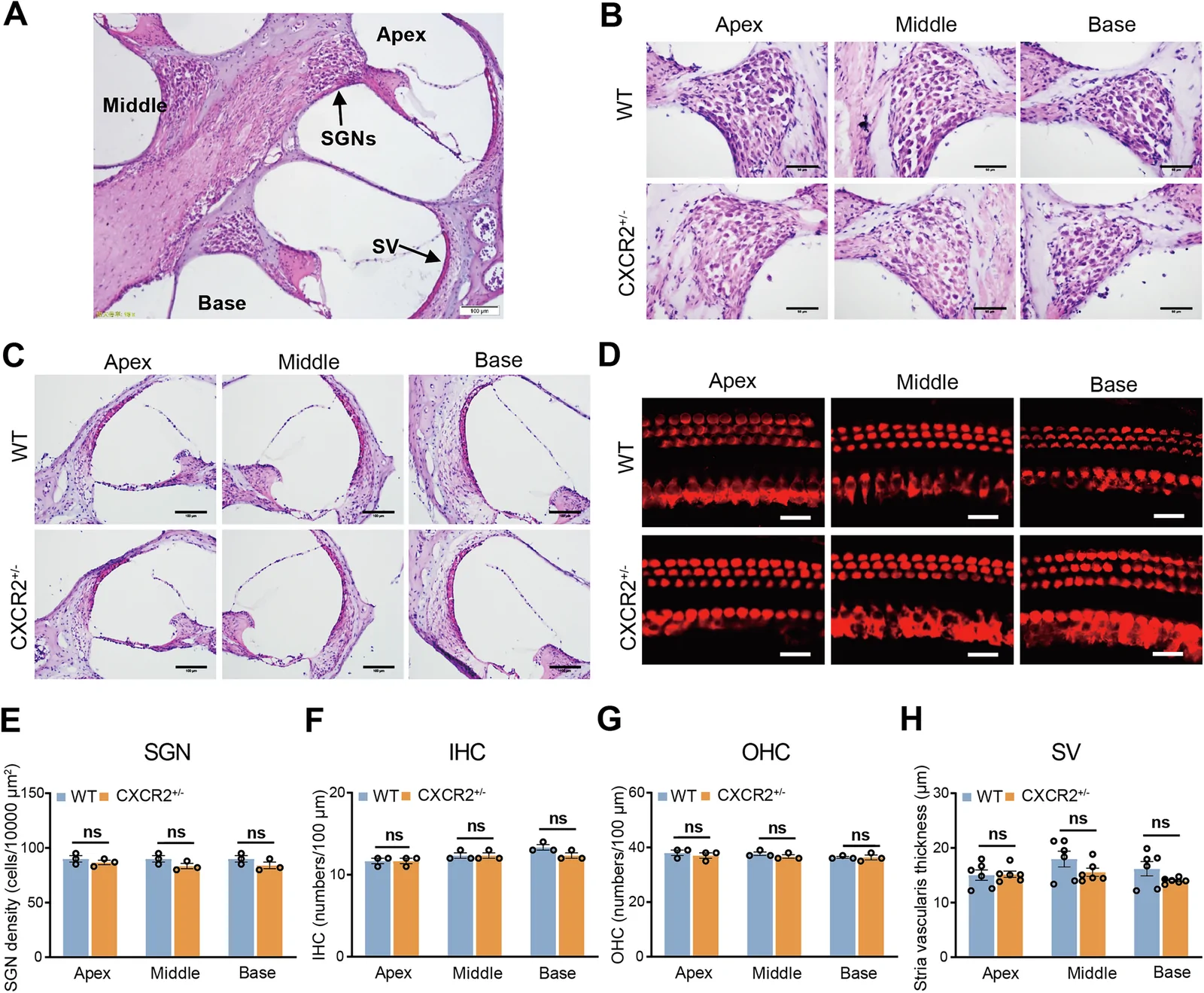
CXCR2^+/−^ mice did not exhibit cochlear degeneration. **A** Cross sections of the whole cochlea were examined by HE staining, showing three regions: apex, middle, and base. Scale bar: 100 μm. **B** Morphometry of SGNs was observed in the apical, middle, and basal regions of the cochlea in 2-month-old WT and CXCR2^+/−^ mice. Scale bar: 50 μm. **C** Morphometry of SV in WT and CXCR2^+/−^ mice, Scale bar: 100 μm. **D** HCs were stained using anti-Myosin 7A in WT and CXCR2^+/−^ mice. Scale bar: 20 μm. **E** Quantification of SGNs at the cochlea’s apical middle and basal regions in WT and CXCR2^+/−^ mice (*n*  =  3 mice). Quantification of inner hair cell (**F**) and outer hair cell (**G**) at the apical, middle, and basal regions of the cochlea in WT and CXCR2^+/−^ mice (*n*  =  3 mice). **H** Quantification of SV thickness in the cochlea of WT and CXCR2^+/−^ mice (*n*  =  6 mice). Data are means ± SEM, *p < 0.05, **p < 0.01, ***p < 0.001. Statistical analyses were performed using two-way ANOVA (**E**–**K**) with a Bonferroni post hoc test.

To investigate the cellular basis of hearing loss induced by CXCR2 deficiency, we subsequently examined the morphology of SGNs, HCs, and SVs in 2-month-old CXCR2^+/−^ mice. HE staining and immunofluorescence showed no apparent morphological abnormalities in SGNs, the stria vascularis, and HCs between WT and CXCR2^+/−^ mice (Fig. 2A–D). Furthermore, the counts of SGNs and HCs revealed that CXCR2^+/−^ cochleae exhibited no cell loss in the apical, middle, and basal turns (Fig. 2E–G). The thickness of stria vascularis did not change in CXCR2^+/−^ mice compared with that in WT mice (Fig. 2H). These data indicate that hearing loss in 2-month-old CXCR2^+/−^ mice may not be caused by cochlear degeneration.

### CXCR2^+/−^ mice exhibit impaired ribbon synapses and altered myelin integrity in SGNs

**Fig. 3:**
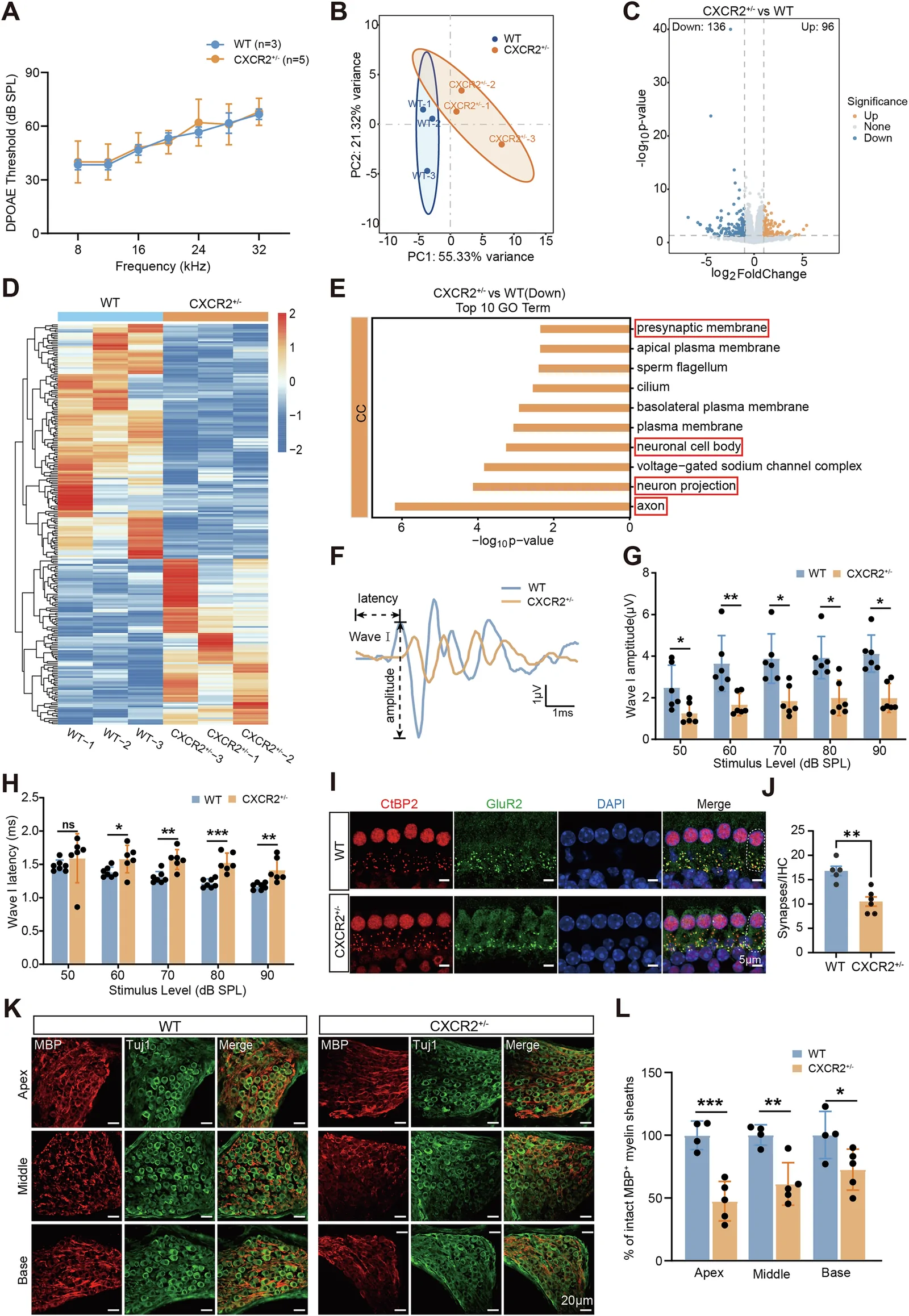
CXCR2 deficiency caused abnormalities of ABR wave I and MBP expression. **A** Distortion product otoacoustic emission (DPOAE) threshold measurement of 2-month-old WT and CXCR2^+/−^ mice. **B** Principal component analysis (PCA) of transcriptomic profiles in cochleae of WT and CXCR2^+/−^ mice. **C** Volcano plots showing DEGs derived from comparisons between CXCR2^+/−^ vs. WT mice. **D** Heat map with hierarchical clustered analysis showing the DEGs identified from panel **C**. **E** Bar charts showing the top 10 enriched key downregulated cellular components identified by Gene Ontology (GO) analysis derived from CXCR2^+/−^ vs. WT mice. **F** Representative ABR waveforms in respond to pure tone stimuli in WT and CXCR2^+/−^ mice. Quantification of ABR wave Ⅰ amplitudes (**G**) and latencies (**H**) in 2-month-old WT and CXCR2^+/−^ mice (*n*  =  6 mice). **I** Immunostaining for CtBP2 and GluR2 in the cochleae of WT and CXCR2^+/−^ mice (*n*  =  3 mice). The area outlined by the white dashed line highlights the synaptic dots located in IHC. Scale bar: 5 µm. **J** Quantification of ribbon synapses from WT and CXCR2^+/−^ mice. **K** Expression of MBP in SGNs from 2-month-old WT and CXCR2^+/−^ mice. Scale bar: 20 μm. **L** Quantitative analysis of SGNs with intact MBP^+^ myelin sheaths in WT (*n*  =  4 mice) and CXCR2^+/−^ mice (*n*  =  5 mice). Data are means ± SEM, *p < 0.05, **p < 0.01, ***p < 0.001. Statistical analyses were performed using two-way ANOVA (**A**, **G**, **H**, **J**, **L**) with a Bonferroni post hoc test.

Next, we explored whether dysfunction of SGNs or HCs resulted in hearing impairments in these mice. First, we evaluated outer hair cell function by measuring the distortion product otoacoustic emissions (DPOAEs), which reflect the cochlear amplification mechanisms [19]. Overall, we found no significant difference in DPOAE thresholds between WT and CXCR2^+/−^ mice (Fig. 3A), indicating that outer hair cell function was unaffected in CXCR2^+/−^ mice.

To further analyze the mechanisms underlying CXCR2 deficiency-induced hearing loss, RNA-Sequencing (RNA-Seq) was conducted as expression profiling. Principal component analysis (PCA) revealed a significant separation of clusters between WT and CXCR2^+/−^ mice (Fig. 3B). Overall, 232 differentially expressed genes (DEGs) were found between WT and CXCR2^+/−^ mice, including 96 up-regulated and 136 down-regulated genes (Fig. 3C). These DEGs are shown in the heatmap in Fig. 3D. Gene ontology (GO) analysis of downregulated DEGs in CXCR2^+/−^ versus WT mice revealed that the most significantly enriched cellular components included the presynaptic membrane, neuronal cell body, neuron projection, and axons (Fig. 3E). This indicates that impaired neuronal function, particularly at the ribbon synapses between hair cells and SGN, may be implicated in this process. As such, we analyzed the ABR wave I amplitude and latency, which specifically monitored inner hair cell-SGN transduction (Fig. 3F–H). CXCR2^+/−^ mice demonstrated reduced wave I amplitude and prolonged latency compared with WT controls. The lower amplitude suggests impaired synchronous firing of the SGN populations (Fig. 3G), whereas the increased latency indicates potential alterations in either myelination or neuronal conduction properties (Fig. 3H).

To determine whether CXCR2 deficiency leads to changes in ribbon synapses, we labeled the presynaptic ribbon protein and postsynaptic receptors with CtBP2 and GluR2 antibodies, respectively (Fig. 3I). Following double labeling, ribbon synapses were classified as the puncta where the two signals colocalized. Quantification revealed that the number of synaptic puncta (CtBP2^+^, GluR2^+^) was significantly reduced in CXCR2^+/−^ mice (Fig. 3J), indicating an impairment of the ribbon synapses. Subsequently, we examined whether CXCR2 deficiency affected SGN myelination by analyzing myelin basic protein (MBP), a key structural component of the myelin sheaths. Immunostaining and quantitative analysis revealed a significant reduction in the proportion of SGNs with intact MBP^+^ myelin sheaths in CXCR2^+/−^ mice compared with WT controls (Fig. 3K, L), indicating compromised myelin integrity. Collectively, these results indicate that CXCR2 deficiency impairs the ribbon synapses and SGN myelination, which may be important causes of hearing impairment.

### SGN excitability is decreased in CXCR2^+/−^ mice

**Fig. 4:**
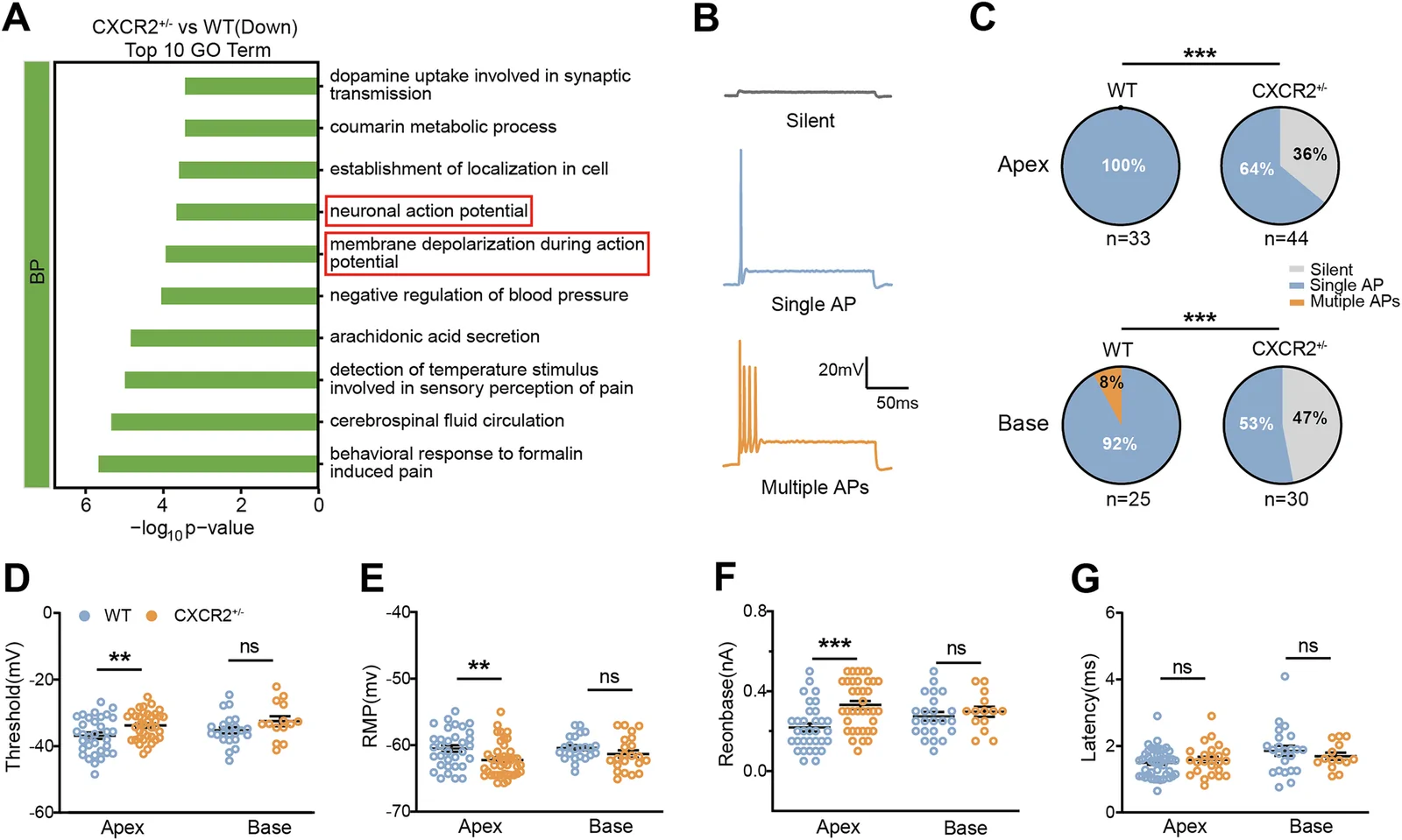
Reduced excitability of SGNs in CXCR2^+/−^ mice. **A** Bar charts showing the top 10 enriched key downregulated biological processes identified by Gene Ontology (GO) analysis derived from CXCR2^+/−^ vs. WT mice. **B** Representative traces show action potential (AP) patterns of SGNs recorded with whole-cell patch clamps. APs were generated by 0.4 nA current injection. **C** Pie charts illustrating the percentage abundance of the different AP patterns of SGNs in WT and CXCR2^+/−^ mice. The AP thresholds (**D**), resting membrane potentials (RMPs) (**E**), rheobases (**F**), and latencies (**G**) were recorded from apical and basal SGNs in WT and CXCR2^+/−^ mice. Data are means ± SEM, *p < 0.05, **p < 0.01, ***p < 0.001. Statistical analyses were performed using Fisher’s exact test (**C**) and two-way ANOVA (**D**–**G**) with Bonferroni post hoc test.

Impairment of ribbon synapses and SGN myelination may lead to dysfunctional action potentials in SGNs. Consistently, GO analysis of DEGs in CXCR2^+/−^ mice (vs. WT) revealed that the enriched down-regulated biological processes included neuronal action potential and membrane depolarization (Fig. 4A). Therefore, we conducted whole-cell patch-clamp recordings to assess the neuronal excitability in WT and CXCR2^+/−^ mice. In WT mice, apical SGNs typically fired single action potentials (APs) in response to a 0.4 nA current injection, whereas basal SGNs exhibited variable firing patterns, including both single and multiple APs (Fig. 4B, C). In contrast, 36.0% of apical and 47.0% of basal SGNs from CXCR2^+/−^ mice failed to produce APs (silent), indicating a markedly reduced excitability (Fig. 4C). In addition, AP thresholds in apical SGNs were significantly higher in CXCR2^+/−^ mice than in WT mice (Fig. 4D). Further, CXCR2^+/−^ mice displayed hyperpolarized resting membrane potentials (RMP) in apical SGNs (Fig. 4E). Rheobase currents were increased in the apical SGNs of CXCR2^+/−^ mice, but no significant difference was observed for the AP latencies between WT and CXCR2^+/−^ mice (Fig. 4F, G). These findings demonstrate that CXCR2 deficiency impairs SGN excitability, which likely contributes to the disruption of auditory signal transmission and subsequent hearing loss.

### Kv1.2 is upregulated in the inner ear of CXCR2^+/−^ mice

**Fig. 5:**
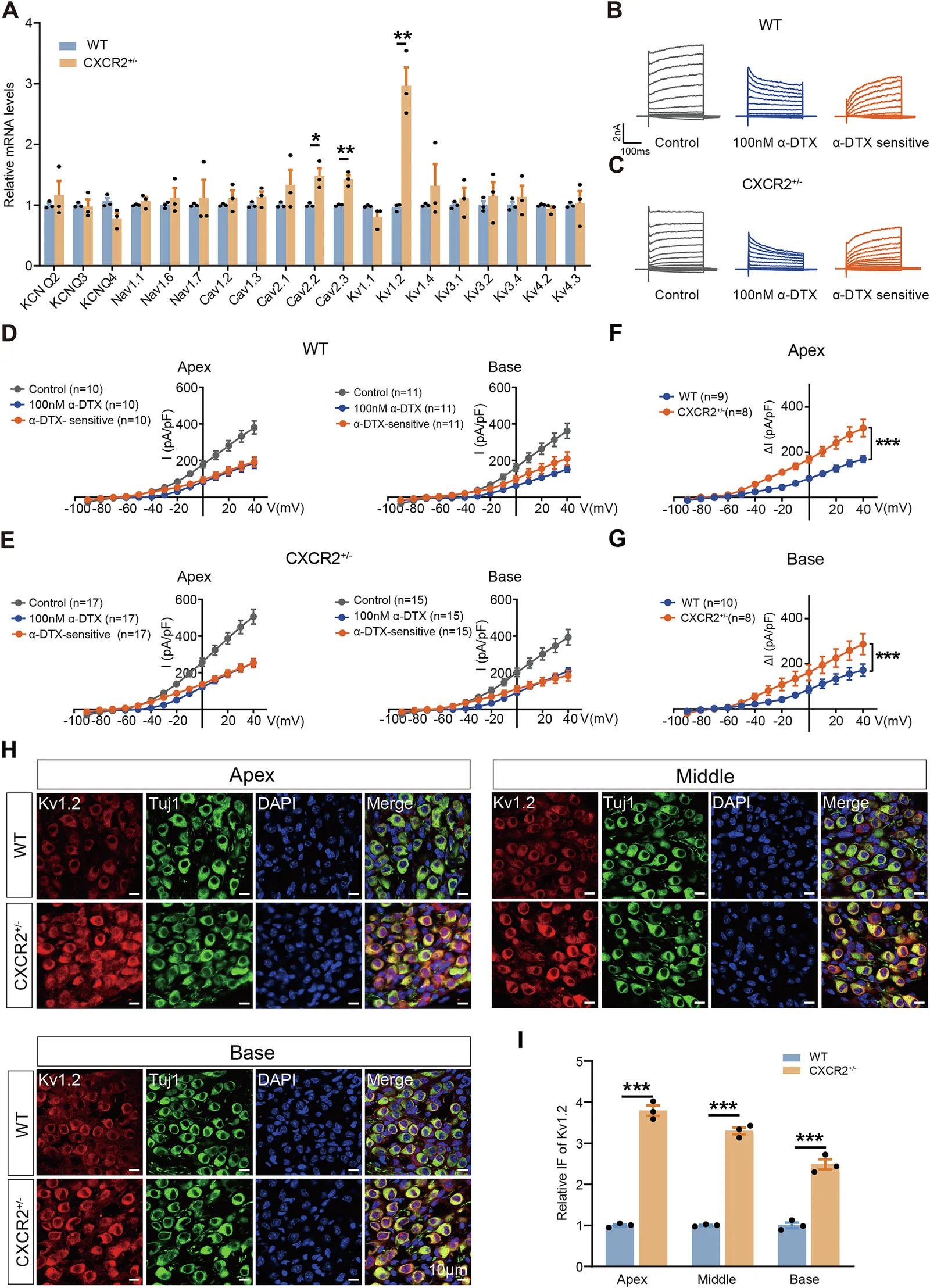
Upregulation of K_v_1 in the SGNs of CXCR2^+/−^ mice. **A** The mRNA expression levels of ion channels in the cochleae of WT and CXCR2^+/−^ mice (*n* = 3 samples, each sample comprising 10 cochleae from 5 mice) were examined by quantitative real-time PCR. **B** Representative current traces recorded from SGNs of WT mice and the resulting traces after application of α-DTX. **C** Representative current traces recorded from SGNs of CXCR2^+/−^ mice and the resulting traces after application of α-DTX. **D** Current density–voltage curves were shown before and after α-DTX (100 nM) application in WT mice. **E** Current density–voltage curves were shown before and after α-DTX (100 nM) application in CXCR2^+/−^ mice. α-DTX-sensitive currents (K_v_1 currents) density was increased in the apical (**F**) and basal (**G**) SGNs of CXCR2^+/−^ mice compared to WT mice. **H** Immunostaining of K_v_1.2 (red) in the SGNs (green) of WT and CXCR2^+/−^ mice. **I **Quantification of K_v_1.2 immunofluorescences in SGNs of WT and CXCR2^+/−^ mice (*n*  =  3 mice). Data are means ± SEM, *p < 0.05, **p < 0.01, ***p < 0.001. Statistical analyses were performed using Student’s unpaired *t*-test (**A**) and two-way ANOVA (**F**, **G**, **I**) with Bonferroni post hoc test.

To determine the cause of decreased SGN excitability in CXCR2^+/−^ mice, we analyzed the ion channel changes in WT and CXCR2 knockout mice. Ion channels are crucial regulators of neuronal excitability, which control resting membrane potential (RMP), action potential threshold, duration, and firing frequency. As such, we sought to identify specific ion channel alterations underlying the reduced SGN excitability in CXCR2^+/−^ mice. Given the established role of specific ion channels in regulating murine SGN excitability [20], we conducted a comparative analysis of their expression profiles in the cochlear tissues of WT and CXCR2^+/−^ mice. Quantitative PCR revealed a significant upregulation of Kv1.2 channel mRNA in CXCR2^+/−^ cochleae (Fig. 5A).

The Kv1.2 channel belongs to the Kv1 family of potassium channels and can assemble into either homomeric or heteromeric tetramers with other Kv1 subunits. To determine whether upregulation of the Kv1.2 channel enhances channel function, we recorded Kv1 currents in SGNs of WT and CXCR2^+/−^ mice. Using a voltage-step protocol ranging from –90 to +40 mV in 10 mV increments from a holding potential of –60 mV, the currents were measured before and after application of the selective Kv1 channel blocker α-dendrotoxin (α-DTX, 100 nM) (Fig. 5B–E). Current density analysis revealed that α-DTX-sensitive Kv1 currents were significantly enhanced in both the apical and basal SGNs of CXCR2^+/−^ mice compared with WT controls (Fig. 5F, G).

As Kv1.1 is also abundantly expressed in SGNs [21, 22] and may form heteromeric channels with Kv1.2, we compared the expression levels of both subunits in WT and CXCR2^+/−^ mice. Immunofluorescence analysis revealed that the expression of Kv1.2, but not Kv1.1, was significantly increased in the SGNs of CXCR2^+/−^ mice (Figs. 5H, I and S3A, B), indicating that Kv1.2 is the primary subunit mediating the increase in Kv1 channel currents.

### Kv1 channel contributes to the excitability regulation of SGNs

**Fig. 6:**
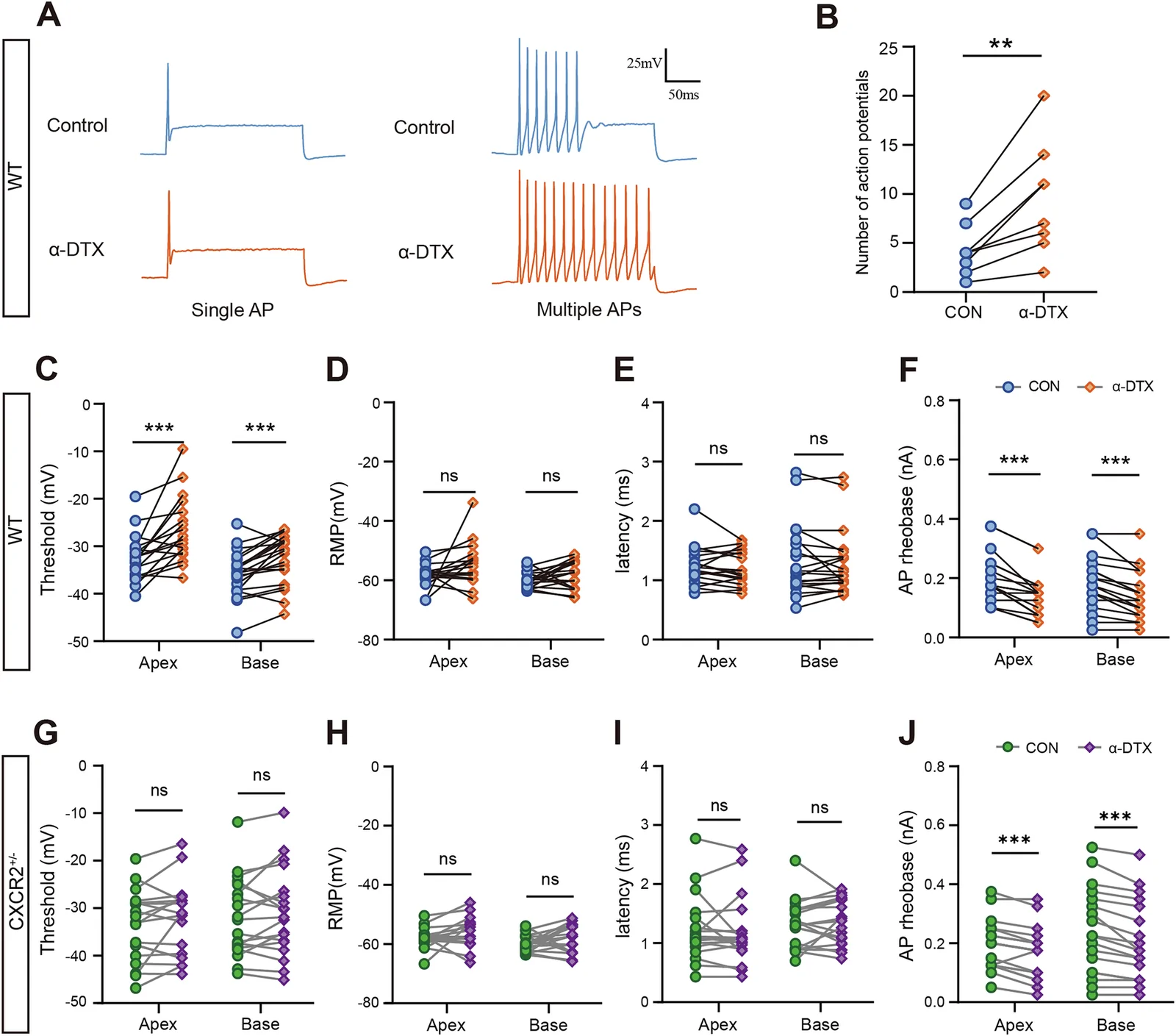
Blockage of the Kv1 channel increased the excitability of SGNs in WT and CXCR2^+/−^ mice. **A** Representative traces of AP recorded in WT SGNs before and after application of α-DTX (100 nM). **B** The AP number of WT SGNs before and after application of α-DTX (100 nM). The AP thresholds (**C**), RMPs (**D**), latencies (**E**), and AP rheobases (**F**) were recorded from apical and basal WT SGNs before and after application of α-DTX (100 nM) (apical: *n* = 18 cells; basal: *n*=21 cells from 3 experiments). The AP thresholds (**G**), RMPs (**H**), latencies (**I**), and AP rheobases (**J**) were recorded from apical and basal CXCR2^+/−^ SGNs before and after application of α-DTX (100 nM) (*n* = 19 cells from 3 experiments). Data are means ± SEM, *p < 0.05, **p < 0.01, ***p < 0.001. Statistical analyses were performed using Student’s paired *t*-test (**B**–**J**).

To verify the effects of Kv1 channels on SGN excitability, we recorded APs before and after applying α-DTX (100 nM) in WT and CXCR2^+/−^ mice. In WT SGNs exhibiting multiple AP patterns, α-DTX treatment significantly increased the number of APs (Fig. 6A, B), demonstrating an enhanced neuronal excitability following Kv1 channel blockade. In contrast, SGNs with single-AP firing patterns showed no response to α-DTX (Fig. 6A, B). AP parameters, including AP thresholds (Fig. 6C), RMPs (Fig. 6D), latencies (Fig. 6E), and AP rheobases (Fig. 6F), were compared between control and α-DTX-treated WT SGNs. AP thresholds were increased in α-DTX-treated SGNs, while the rheobase currents were decreased (Fig. 6C, F). No significant differences were observed in RMP or latency (Fig. 6D, E).

Moreover, we recorded the APs in CXCR2^+^^/^^−^ mice following α-DTX application (100 nM) (Fig. 6G–J). Although no significant differences were observed in the threshold, RMP, or latency (Fig. 6G–I), the rheobase currents were found to be decreased following α-DTX treatment (Fig. 6J), indicating increased excitability. Taken together, these results indicate that Kv1 blockade partially restores SGN excitability in CXCR2^+^^/^^−^ mice, likely by upregulating Kv1.2.

### CXCR2 knockdown in SGNs specifically recapitulates hearing loss

**Fig. 7:**
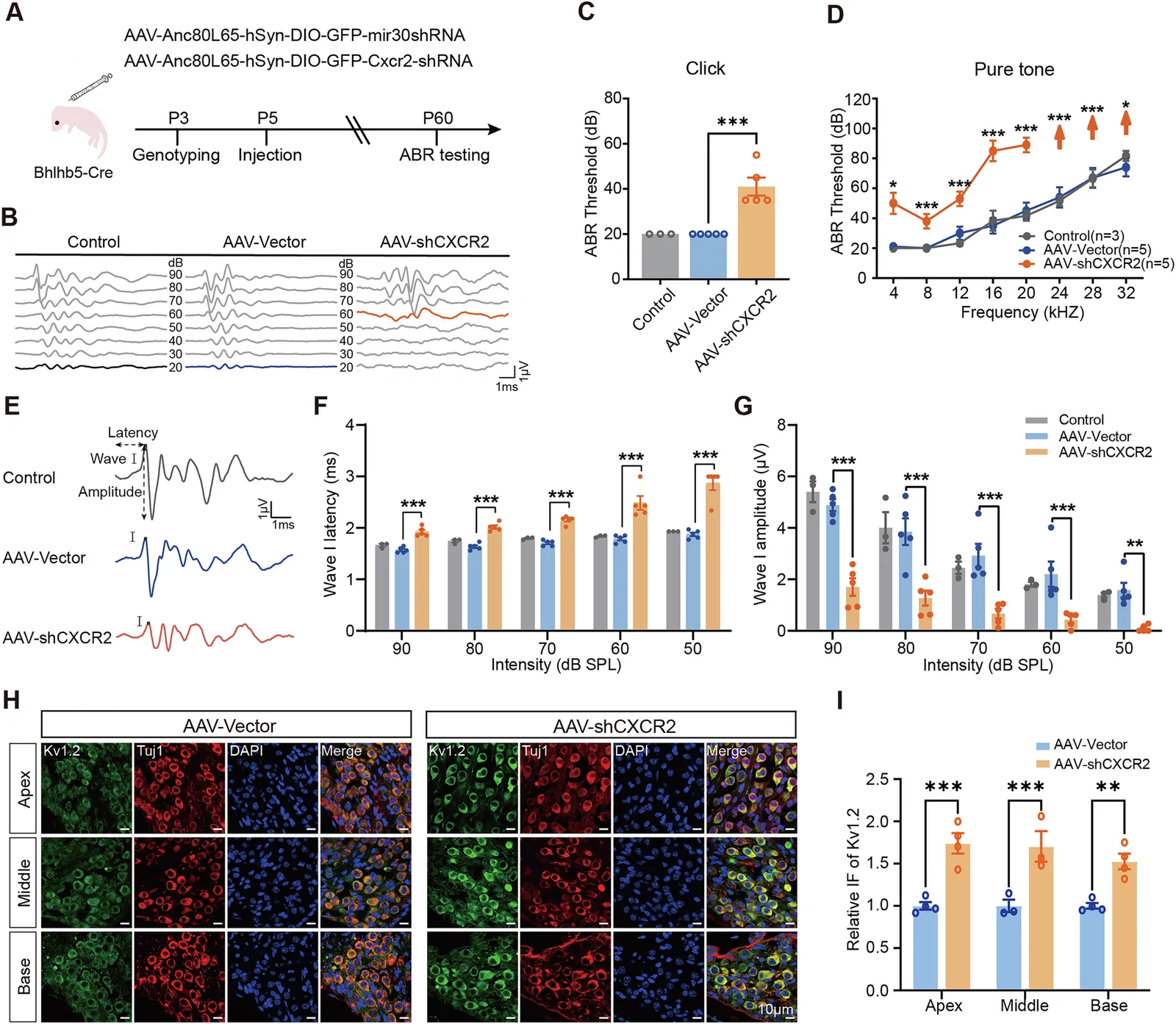
Knockdown of CXCR2 in SGNs caused hearing loss in WT mice. **A** Timeline for knockdown experiment in *Bhlhb5-Cre* mice. Mice in the AAV-Vector group were injected with AAV-Anc80L65-hSyn-DIO-GFP-mir30shRNA (Vector) in the ear. Mice in the AAV-shCXCR2 group were injected with AAV-Anc80L65-hSyn-DIO-GFP-*Cxcr2*-mirshRNA (AAV-shCXCR2) in the ear. **B** Representative ABR waveforms respond to click stimuli in the mice in control, AAV-Vector, and AAV-shCXCR2 groups. ABR thresholds to click (**C**) and pure tone stimuli (**D**) in control AAV-Vector and AAV-shCXCR2 groups. **E** Representative ABR waveform Ⅰ in response to pure tone stimuli in control, AAV-Vector, and AAV-shCXCR2 mouse groups. Quantification of ABR wave Ⅰ latencies (**F**) and amplitudes (**G**) in control (*n*  =  3 mice), AAV-Vector (*n*  =  5 mice), and AAV-shCXCR2 (*n*  =  5 mice) mouse groups. **H** Immunostaining of Kv1.2 (green) in the SGNs (red) of AAV-Vector and AAV-shCXCR2 treated mice. Scale bar: 10 μm. **I** Quantification of Kv1.2 immunofluorescence showed increased Kv1.2 expression in SGNs from apical, middle, and basal regions of the cochlea in AAV-shCXCR2 group mice (*n*  =  4 mice). Data are means ± SEM, *p < 0.05, **p < 0.01, ***p < 0.001. Statistical analyses were performed using one-way ANOVA (**C**) and two-way ANOVA (**D**, **F**, **G**, **I**) with a Bonferroni post hoc test.

To determine whether CXCR2 deficiency in SGNs contributes to hearing loss, we delivered AAV-expressing *Cxcr2* shRNA to *Bhlhb5-Cre* mice at P5 to specifically knockdown CXCR2 in SGNs (Fig. 7A). ABR measurements were conducted at P60 (Fig. 7A). In the AAV-vector group, *Bhlhb5-Cre* mice were injected with AAV-Anc80L65-hSyn-DIO-GFP-mir30shRNA (AAV-vector) into the posterior semicircular canal, while mice in the AAV-shCXCR2 group were injected with AAV-Anc80L65- hSyn-DIO-GFP-Cxcr2-mirshRNA (AAV-shCXCR2). The efficiency of CXCR2 knockdown was examined by immunostaining of AAV-shCXCR2 mice at P60 (Fig. S4). Compared with the AAV-vector group, AAV-shCXCR2 mice exhibited significantly increased ABR thresholds, indicating hearing impairment (Fig. 7B–D). Moreover, increased latency and reduced amplitude of ABR wave I were observed in the AAV-shCXCR2 group, suggesting that CXCR2 knockdown in SGN impairs auditory signal transduction (Fig. 7E–G). Additionally, Kv1.2 expression was upregulated in the AAV-shCXCR2 mice (Fig. 7H, I). These findings indicate that CXCR2 in SGNs is essential for normal hearing.

## Discussion

CXCR2 was first identified in 1992 as a high-affinity receptor for CXCL8/IL-8 [23]. Further investigations showed that CXCR2 is activated by a variety of CXC chemokines, including CXCL1, CXCL2, CXCL3, CXCL5, CXCL6, CXCL7, and CXCL8 [7]. This promiscuous binding capacity enables CXCR2 to orchestrate diverse biological processes, ranging from immune cell trafficking to tissue homeostasis and repair. However, systematic studies investigating the role of CXCR2 in the auditory system are lacking. The present study characterized the expression and distribution of CXCR2 in the cochlea, showing that CXCR2 deficiency leads to hearing impairment via a novel mechanism involving Kv1.2 upregulation and subsequent reduction of SGN excitability. SGN-specific CXCR2 knockdown recapitulated hearing deficits, indicating that CXCR2 in SGNs plays a critical role in maintaining normal hearing.

### CXCR2 is expressed in multiple cell types in the cochlea

In mammals, the 20 types of chemokine receptors are divided into four subfamilies: CCR−, CXCR−, XCR−, and CX3CR− [24–26]. Upon activation, these receptors transmit extracellular chemical signals via intracellular pathways to regulate cellular functions [24, 25]. Within the cochlea, several chemokine receptors, including CX3CR1, CCR2, and CCR7, have been identified as participating in various physiological and pathological processes [27]. For example, CX3CR1 in macrophages plays a protective role because its deletion causes irreversible synaptic damage, in addition to SGN and HC injuries, following noise exposure [28]. Macrophage CCR2 and CCR7 appear to mediate noise- and drug-induced hearing loss through inflammatory regulation [29, 30]. Additionally, CXCR2 has also been found to be expressed in macrophages and may mitigate cisplatin-induced ototoxicity [18]. These findings indicate that chemokine receptors in the cochlea function primarily via immunomodulatory mechanisms.

Notably, CXCR2 is a member of the G protein-coupled receptor (GPCR) family and is broadly expressed not only in macrophages, but also in various non-immune cell types, such as neurons and smooth muscle cells [8–10, 13]. However, its expression pattern and functional significance in non-macrophages within the cochlea currently remain unknown. Our results indicate that CXCR2 is present in SGNs and may contribute to the regulation of auditory signaling. In addition, CXCR2 expression was detected in HCs and stria vascularis, the functions of which require further investigation.

### CXCR2 deficiency causes hearing loss

To investigate the role of CXCR2 in hearing, we assessed auditory function in CXCR2-deficient mice. We found that both CXCR2^+/−^ and CXCR2^−/−^ mice at 2 months of age exhibited hearing impairments. These results indicate that CXCR2 may be crucial for maintaining cochlear function and that other chemokine receptors may be unable to fully compensate for reduced CXCR2 expression. Consequently, heterozygous mice retain residual CXCR2 but still develop a deafness phenotype.

Notably, given the wide expression of CXCR2, the hearing loss observed in CXCR2^+/−^ mice cannot completely exclude the potential contribution of CXCR2 deficiency in central auditory neurons; this is a notable limitation of this study. To identify the target cells of CXCR2, we employed a genetic knockdown approach by injecting AAV-expressing *Cxcr2* shRNA into *Bhlhb5*-Cre mice to selectively knockdown CXCR2 in SGNs. Our results indicated that SGN-specific CXCR2 knockdown recapitulated the hearing loss phenotype observed in CXCR2^+/−^ mice. Together with the findings of abnormal ABR wave I and normal DPOAE in CXCR2^+/−^ mice, these data strongly support that the auditory dysfunction caused by CXCR2 deletion primarily originates from the peripheral auditory system. Furthermore, combined with the observed reduction in SGN excitability and myelin alterations, the present findings indicate that CXCR2 in SGNs plays a critical role in mediating hearing. Using SGN-specific conditional knockout mice remains a more rigorous approach.

### CXCR2 is crucial for SGN excitability

CXCR2 functions can vary according to cell type. In nociceptive sensory neurons, the CXCL5-activated CXCR2 induces gout, pain, and inflammation [31]. In murine neutrophils, CXCR2 suppresses the IL-17A/G-CSF axis that regulates neutrophil homeostasis [32]. Additionally, CXCR2 in tumor progenitor cells controls transcriptional programs that facilitate melanoma growth [33]. In the present study, we revealed a previously unrecognized role of CXCR2 in auditory function; specifically, our RNA-seq data showed the enrichment of DEGs associated with synapses, neuronal processes, and axons, implicating SGNs as potential targets of CXCR2 deficiency. Although the data were obtained from whole cochlear tissues and could not resolve SGN-specific changes, these clues led us to further examine the function of the SGN. CXCR2 deficiency decreased SGN excitability and increased Kv1 channel currents. Kv1 channels negatively regulate neuronal excitability, with their upregulation inhibiting action potential firing and downregulating neuronal activity [22, 34]. Consistently, our electrophysiological data revealed that inhibition of the Kv1 channel by α-DTX increased SGN excitability. This finding further confirms that maintenance of SGN excitatory function by CXCR2 is associated with Kv1 channels.

The Kv1 channel family belongs to voltage-gated potassium channels and comprises eight subunits (Kv1.1–Kv1.8). Functional Kv1 channels assemble as tetramers with four subunits, which can form homomeric or heteromeric combinations. Previous studies have identified the expression of the Kv1.1, Kv1.2, and Kv1.4 subunits in SGNs [21, 22, 35], which aligns with our mRNA profiling of mouse cochlear tissue. To characterize the electrophysiological properties of Kv1 channels in SGNs, we recorded Kv1 currents following the application of α-DTX. Notably, α-DTX selectively inhibits Kv1.1, Kv1.2, and Kv1.6 subunits [34]. Since Kv1.6 expression has not been reported in SGNs, we concluded that the recorded α-DTX-sensitive current (Kv1 current) is primarily mediated by Kv1.1 and Kv1.2 subunits.

Immunofluorescence staining revealed that in the SGNs of CXCR2^+/−^ mice, Kv1.2 expression was significantly upregulated, while Kv1.1 levels remained unchanged. Combined with the observed changes in electrophysiological recordings, these results indicate that the increased Kv1 current in CXCR2^+/−^ mice may result from Kv1.2 subunit upregulation, indicating that Kv1.2 may be a key regulator of SGN excitability. However, given its broad expression pattern, whether Kv1.2 exerts similar functions in other cochlear cell types remains to be determined. Similarly, the precise molecular mechanisms by which CXCR2 signaling modulates Kv1.2 expression and function.

In addition, we observed reduced ribbon synapse numbers and altered myelin integrity in CXCR2-deficient mice. Whether these changes are directly linked to Kv1.2 upregulation remains unclear; however, they may collectively contribute to defective auditory signal transmission. The mechanisms underlying the CXCR2-mediated regulation of these structures warrant further investigation.

**Fig. 8:**
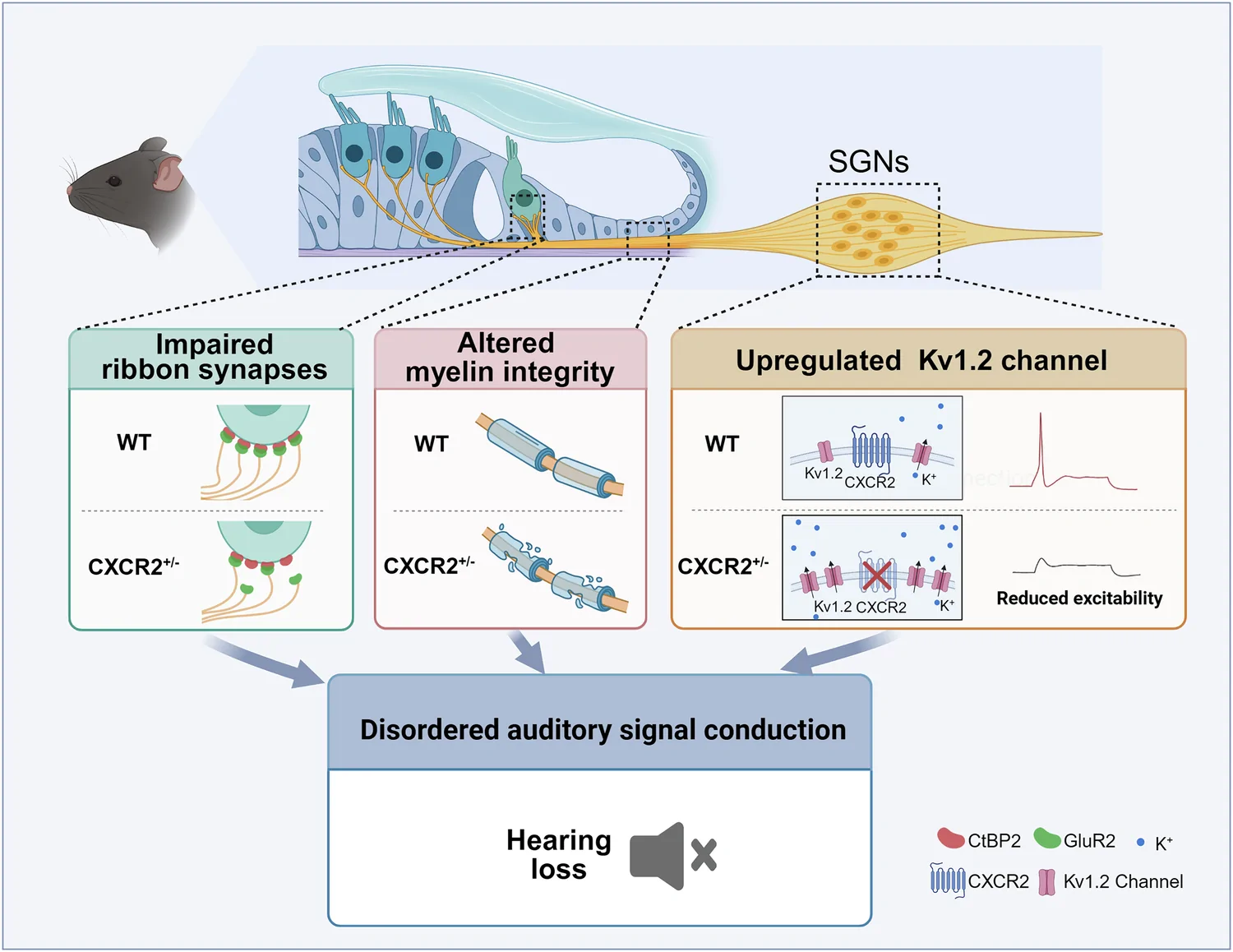
Schematic depicting the proposed role of CXCR2 in hearing. In CXCR2^+/−^ mice, SGNs displayed three key pathological changes: upregulation of the Kv1.2 potassium channel, ribbon synapse damage, and altered myelin integrity. These deficits collectively disrupted auditory signal conduction, resulting in hearing loss in the mice.

In conclusion, our findings reveal that CXCR2 is a key regulator of SGN auditory function. The Kv1.2-mediated reduction in SGN excitability, together with synaptic loss and myelin impairment, likely contributes to hearing loss in CXCR2^+/−^ mice. (Fig. 8) These results establish a novel noncanonical function of CXCR2 in neural excitability, extending beyond its well-characterized roles in immunity and inflammation.

## Materials and methods

### Animals

C57BL/6 mice were obtained from Charles River Laboratories. CXCR2 knockout mice were obtained from the Institute of Laboratory Animal Sciences of the Chinese Academy of Sciences. *Bhlhb5-Cre* mice were a gift from Dr. Renjie Chai [36, 37] (Southeast University). Mice of both sexes were used in this study. PASS Sample Size Software was used to evaluate the sample size. The experimental mice were grouped by a simple random sampling method, and the relative assays were performed blindly. All mice were bred under a standard 12:12-h light–dark cycle with the room kept at approximately 22 °C. Mouse tail genotyping was performed using the primers listed in Table S1.

### ABR and DPOAE measurement

ABR measurements were performed as previously described [38]. Briefly, mice were anesthetized with ketamine (100 mg/kg) and xylazine (10 mg/kg) via intraperitoneal injection. Platinum needle electrodes were inserted subcutaneously into the vertex of the head (reference), the ipsilateral mastoid (recording), and the contralateral hind legs (ground). The ABR was measured using a TDT system (Tucker-Davis Technologies, Gainesville, FL, USA). Hearing threshold was defined as the lowest intensity required to generate a reproducible ABR waveform. The responses were measured for clicks and pure tones at frequencies of 4–32 kHz. The stimulus levels of sound were presented from 90 to 20 dB sound pressure level (SPL) in increments of 5 dB SPL. Latencies of ABR wave I were measured using a 90 dB pure tone. The amplitudes were calculated as peak-to-peak amplitudes of the next negative trough.

DPOAE at 2*f*1−*f*2 was elicited from the test mice using BioSig-RP software and a TDT system (Tucker-Davis Technologies). 2*f*1−*f*2(*f*2/*f*1 = 1.2) was measured at different frequencies to predict auditory thresholds. Hearing thresholds were defined as the level at which 2*ƒ*1−*ƒ*2 exceeded the noise floor by at least 6 dB.

### Cochlear whole mount preparation

Cochleae were isolated and perfused with 4% paraformaldehyde through the round and oval windows and kept overnight at 4 °C. Then cochleae were decalcified in 10% ethylenediaminetetraacetic Acid (#798681, Sigma, Darmstadt, Germany) in phosphate-buffered saline (PBS) for 2–3 days at 4 °C. After immersion in a graded sucrose series (10% and 30%) overnight, the cochleae were embedded in OCT compound (Tissue-Tek) and sectioned at 10 μm for study.

### Cell count

Cochlear sections were stained with hematoxylin and eosin (H&E) to evaluate SGN morphometry and density. The Rosenthal Canal is divided into three regions: apex, middle, and base. SGN densities in these three regions were measured using Image-Pro Plus 5.1. The cells in one field (apex, middle, or base) were counted in each section, and six representative sections were analyzed in one cochlea per mouse. Cells displaying Myosin 7A labeling were quantified as the number of positive cells per 100 μm of basilar membrane length from the apex, middle, and base for HC counts.

### Immunofluorescence

The specimens were permeabilized in 0.1% Triton X-100 and incubated in 10% serum for 60 min at room temperature followed by incubation with primary antibodies: mouse anti-Tuj1 (#801202, Biolegend, CA, USA,), rabbit anti-CXCR2 (#20634-1-AP, Proteintech, USA), rabbit anti-Kv1.1 (#APC-009, Alomone labs, Israel), mouse anti-CtBP2 (#612044, BD Biosciences), mouse anti-GluR2 (#MAB397, Millipore), rabbit anti-Kv1.2 (#APC-010, Alomone labs, Israel), rabbit anti-MBP (#AB218011, Abcam, USA) and rabbit anti-Myo7a (#PT-25-6790, Proteus, Ramona, CA, USA), overnight at 4 °C. The specimens were rinsed with PBS three times and incubated with goat anti-mouse (IgG1)-AF568 (#A21124, Invitrogen), goat anti-mouse (IgG2a)-AF488 (#A21131, Invitrogen), Alexa 488-conjugated and Alexa 568-conjugated secondary antibodies (#115-545-003, 115-165-003, 111-545-003,111-165-003, Jackson Immuno Research, PA, USA; 1:400) for 1.5 h at room temperature. Nuclei were counterstained with 4,6-diamidino-2-phenylindole dihydrochloride (DAPI; #D9542; Sigma-Aldrich) for 10 min at room temperature. Samples were then mounted in ProLong™ Gold Antifade Mount (# P36934, Invitrogen, CA, USA). Images were captured using Leica LAS AF Lite and processed using ImageJ and Photoshop CS5 (Adobe, San Jose, CA, USA). The number of synaptic ribbons puncta (CtBP2^+^, GluR2^+^) was measured using the 3D-Objects Counter Plugin for Images.

### Quantitative real-time polymerase chain reaction (qPCR)

Total RNA samples from the mouse cochlea were extracted using the RNAeasy Micro Kit (Qiagen, Hilden, Germany). RNA was reverse transcribed using PrimeScript reverse transcriptase (#RR036Q; TaKaRa Bio). Quantitative PCR was performed using a TB GREEN Kit (Takara Bio Inc., China) with a two-step cycling program on a Bio-Rad CFX Connect Real-Time PCR System. Relative fold of differences in expression was calculated using the formula: 2^−ΔΔCt^ after normalization to GAPDH expression. All primers used in this study were designed using Primer Premier 6.25 (PREMIER Biosoft International, CA, USA) and synthesized by Sangon Biotech (Shanghai, China). The primer sequences are shown in Table S2.

### RNA sequencing

Cochlear total RNA was extracted from 2-month-old (2M) mice (5 mice per sample) using TRIzol reagent (Invitrogen, Carlsbad, CA, USA). RNA purification, quantification, and reverse transcription were subsequently performed by OE Biotech Co., Ltd. (Shanghai, China). Then the libraries were constructed using the VAHTS Universal V10 RNA-seq Library Prep Kit (Premixed Version). The prepared cDNA libraries were sequenced on a DNBSEQ-T7 platform (BGI Genomics, Shenzhen, China), generating 150 bp paired-end raw reads. Raw reads in FASTQ format were first subjected to quality control using fastp software. Low-quality reads were removed to obtain high-quality clean reads for subsequent analysis. Differential gene expression analysis was conducted using the DESeq2 R package. Genes with a *Q*-value < 0.05 and a fold change (FC) > 2 or FC < 0.5 were defined as significantly differentially expressed genes (DEGs). Functional enrichment analyses of DEGs, including Gene Ontology (GO), Kyoto Encyclopedia of Genes and Genomes (KEGG) pathway, were performed separately based on the hypergeometric distribution using R software (version 3.2.0). Significantly enriched terms were screened based on statistical significance. Column charts for visualizing the enrichment results were generated using R (v 3.2.0). RNA sequencing of the raw data and processed data for this study have been deposited in the Sequence Read Archive (PRJNA1498831).

### SGN culture

The SGNs were isolated from the mouse inner ear following a previously described procedure [39]. Mice were euthanized at 2 months of age, and the temporal bones were removed. The dissecting solution consisted of minimum essential medium with Hank’s Balanced Salt Solution (Invitrogen) supplemented with 0.2 g/L kynurenic acid, 10 mM MgCl_2_, 2% fetal bovine serum (FBS; v/v), and 6 g/L glucose. The SGN tissue was divided into two segments: the apex and base. The two-segment tissue was subjected to separate digestion in an enzyme mixture comprising collagenase type I (1 mg/mL) and DNase (1 mg/mL) at 37 °C for 15 min. Following gentle trituration and centrifugation (2000 rpm for 5 min) in 0.45 M sucrose, the cell pellets were reconstituted in culture media (Neurobasal^TM^ A, supplemented with 2% B27 (v/v), 0.5 mM L-glutamine, 100 units/mL penicillin; Invitrogen). The SGN suspension was then filtered and plated onto pretreated glass coverslips. The SGNs were maintained in culture for a period of 24–48 h prior to electrophysiological recordings, allowing for the detachment of Schwann cells from the soma.

### Western blot

Cochlear tissues (4–5 mice were used for each sample) were harvested and homogenized in RIPA lysate containing a protease inhibitor cocktail. Protein concentration was determined using a BCA Protein Assay Kit (Solarbio, China). Equal amounts of protein were resolved by SDS–PAGE and transferred to a PVDF membrane (Millipore, USA). Membranes were blocked with 1× TBST containing 5% dry milk for 2 h at room temperature and probed with anti-CXCR2 (#20634-1-AP, Proteintech, 1:1000) and anti-GAPDH (#60004-1, Proteintech, 1:5000) overnight at 4 °C. Then, the membranes were incubated with secondary antibodies (IRDye 800CW goat anti-rabbit, 1:10,000; IRDye 800CW goat anti-mouse, 1:10,000; LI-COR, Biosciences) for 90 min at room temperature. The immunoreactive bands were quantified using Image Lab 4.0 (Bio-Rad, USA).

### Electrophysiology

Whole-cell voltage-clamp recordings of K^+^ currents in SGNs were performed using a Multiclamp 700 B amplifier (Molecular Devices, Sunnyvale, CA, USA). Signals were filtered at 2 kHz with a low-pass Bessel filter and digitized at ≥20 kHz using a 12-bit acquisition system, Digidata 1332 (Axon Instruments), and pClamp 10.7 software (Molecular Devices). Fire-polished electrodes (3–4 MΩ) were pulled from the borosilicate glass. The electrodes contained (in mM) 140 KCl, 1 MgCl_2_, 0.1 CaCl_2_, 10 4-(2-hydroxyethyl)-1-piperazineethanesulfonic acid (HEPES), 1 EGTA, 4 K_2_ATP, and 0.5 Na-GTP, adjusted to a pH of 7.35 with potassium hydroxide. The bath solution contained (in mM) 130 NaCl, 0.5 MgCl_2_, 2 CaCl_2_, 10 HEPES, and 10 glucose, adjusted to pH 7.4 with NaOH. For recording K^+^ current in a whole-cell configuration, SGN were held at −70 mV with 300 ms step voltages from −90 to 40 mV (Δ*V* = 10 mV). The Kv1 currents were assessed as α-DTX (GLPBIO, USA)-sensitive currents. The current magnitude was divided by the cell capacitance (pF) to generate the current density–voltage relationship. Action potentials were measured using the same electrodes and bath solutions used in the Kv1 current-recording experiment. Action potentials were evoked by injection of 0.4 nA current in SGNs in whole-cell current-clamp recordings.

### Virus injection

*Bhlhb5-*Cre neonatal mice (P5) were used for AAV treatment experiments. A postauricular incision was made behind the auricle, followed by the exposure of the posterior semicircular canal and lateral semicircular canal. The AAV-Vector group received injections of AAV-Anc80L65-hSyn-DIO-GFP-mir30shRNA, whereas the AAV-CXCR2 group was injected with AAV-Anc80L65-hSyn-DIO-GFP-Cxcr2-shRNA (targeting Gene ID: 12765; Weizhen, China). The control mice remained untreated. The ABR measurements were performed at P60.

### Statistical analysis

All data are presented as the mean ± SEM. Statistical analyses were performed using GraphPad Prism (version 8.0.2; GraphPad Software, Inc., San Diego, CA, USA). The Student's *t*-test was used to compare data between the two groups, and ANOVA was used for multiple comparisons. The Kruskal–Wallis test was used for comparisons of data with non-normal distributions or heterogeneity of variance. *, **, and *** indicated statistically significant results compared to appropriate controls and indicated *p* < 0.05, *p* < 0.01, and *p* < 0.001, respectively.

## Supplementary information

Supplemental Figure

Table

Original western blots

## Data Availability

All data generated or analyzed during this study were included in the manuscript.
