# Supplementary material for: CXCR2 deficiency impairs auditory function in spiral ganglion neurons by altering excitability
Source: Cell Death Discov. 2026 Oct 15;12:432. doi: 10.1038/s41420-026-03366-7 (PMC13720065; doi:10.1038/s41420-026-03366-7)
Supplement: Supplementary file 1 [file 41420_2026_3366_MOESM1_ESM.docx]

**Supplement**

**Fig. S1 Expression of CXCR2 in SGNs during cochlea development**

**(A)** Confocal images showing the CXCR2 expression in SGNs of WT mice at different developmental stages. SGNs were indicated by Tuj1 (red) immunoreactivity. Scale bar: 10 μm. (**B**) Quantification of CXCR2 fluorescence intensity in SGNs at different developmental stages (n=3 mice).

**Fig. S2 Expression of CXCR2 in WT and CXCR2 knockout mice**

**(A)** PCR genotyping of WT and CXCR2 knockout mice using genomic DNA prepared from tail biopsies. (**B**) Western blot using an anti-CXCR2 antibody was performed to examine CXCR2 expression in WT and CXCR2^+/-^ mice. GAPDH was included as an internal control. (n=3 samples). (**C**) The mRNA expression levels of CXCR2 in the cochleae of WT and CXCR2^+/-^ mice were examined by quantitative real-time PCR. (n=3 samples) (**D, E**) Confocal images showing the CXCR2 expression in SGNs (D) and hair cells (E) in the cochlea of WT, CXCR2^+/-^ and CXCR2^-/-^. Scale bar: 10 μm.

**Fig. S3** **K_v_1.1 expression showed no significant change in SGNs of CXCR2^+/-^ mice.** (**A**) Immunostaining of K_v_1.1 (green) in the SGNs (red) of WT and CXCR2^+/-^mice. (**B**) Quantification of K_v_1.1 expression in the SGNs of WT and CXCR2^+/-^mice (n=3 mice). Scale bar: 10 μm. Data are means ± SEM.

**Fig. S4 Expression of CXCR2 in AAV treated mice. (A)** CXCR2 expression in SGNs from AAV-Vector and AAV-shCXCR2 group mice. (**B**) Quantification of CXCR2 expression in the SGNs of AAV-Vector and AAV-shCXCR2 mice (n=4 mice). Scale bar: 10 μm. Data are means ± SEM, ****p*<0.001.
