# Supplementary material for: CXCR2 deficiency impairs auditory function in spiral ganglion neurons by altering excitability
Source: Cell Death Discov. 2026 Oct 15;12:432. doi: 10.1038/s41420-026-03366-7 (PMC13720065; doi:10.1038/s41420-026-03366-7)
Supplement: Supplementary file 2 [file 41420_2026_3366_MOESM2_ESM.docx]

**Table S1. The primer sequences for genotyping**

| Primer | Sequence (5′-3′) |
| --- | --- |
| CXCR2 WT forward | AGAATTGGGGTCAGGGTACA |
| CXCR2 WT reverse | CCCTTAGGCTGCAAATGAAC |
| CXCR2 KO forward | CTTGGGTGGAGAGGCTATTC |
| CXCR2 KO reverse | AGGTGAGATGACAGGAGATC |

**Table S2. The primer sequences for quantitative real-time PCR**

| Primer | Sequence (5′-3′) |
| --- | --- |
| Kv7.2 forward | AGGAAGCCGTTCTGTGTGAT |
| Kv7.2 reverse | GCAGAGGAAGCCAATGTAC |
| Kv7.3 forward | GAAGACAGGGGCTATGGGAAT |
| Kv7.3 reverse | GTTTTGGAGTGGATGGAGGTC |
| Kv7.4 forward | ATGGGGCGCGTAGTCAAGGT |
| Kv7.4 reverse | GGGCTGTGGTAGTCCGAGGTG |
| Nav1.1 forward | CTCCTCAAATGGGTGGCCTA |
| Nav1.1 reverse | GATTTGATGGCCCCGAGTTC |
| Nav1.6 forward | CCTGGTGTTCACTGGGATCT |
| Nav1.6 reverse | AGGCCCAGCTCCATTAAACT |
| Nav1.7 forward | CGAGAGCGGAGAGATGGATT |
| Nav1.7 reverse | TTCGCTTCAGTGTGGTTGTG |
| Cav1.2 forward | CGGTTACTTTAGTGATCCCTGG |
| Cav1.2 reverse | CGGGATTAGTCTCACTGAGAATG |
| Cav1.3 forward | TTTGACAATGTCCTTTCGGCT |
| Cav1.3 reverse | TTCTCACCGTTTGAATCAATAGCT |
| Cav2.1 forward | CATCATCATCGGCTCCTTTT |
| Cav2.1 reverse | GAAAAGCTCTCCGGTTCTCC |
| Cav2.2 forward | TAGACTTGGGCTTGCTGCTG |
| Cav2.2 reverse | CTCGAGAACGCAAATGCCAC |
| Cav2.3 forward | AACCCACTTCAACACCCACG |
| Cav2.3 reverse | GCACGATCTGCAGGCTAGGT |
| Kv1.1 forward | GGCGAGAGGGGTTTCCAAAT |
| Kv1.1 reverse | GGCTTCAGAGCCAGAAGGTT |
| Kv1.2 forward | GGAGGCTCTGGTACCCATCT |
| Kv1.2 reverse | CCTTTGGAAGGAAGGAGGCA |
| Kv1.4 forward | ACCACCATCATCAGACACGT |
| Kv1.4 reverse | GCCTGTGGTGGAGTTTCTTC |
| Kv3.1 forward | CTTATCAACCGGGGAGTACG |
| Kv3.1 reverse | AATGACAGGGCTTTCTTTGC |
| Kv3.2 forward | TTACACGTGCGCTTCTGATG |
| Kv3.2 reverse | CAGAGGACACGGAGAGTTGT |
| Kv3.4 forward | GCCTTTGCCTCTCTCTTCTT |
| Kv3.4 reverse | GGTGGATCTCCGTCACATTT |
| Kv4.2 forward | CCCATCAAGTCACAGTCCCT |
| Kv4.2 reverse | CCCGACACATTGGCATTAGG |
| Kv4.3 forward | TTCTTTTGCCTGGACACTGC |
| Kv4.3 reverse | GCCACAACGTCGATAATGCT |
