# Supplementary material for: CXCR2 deficiency impairs auditory function in spiral ganglion neurons by altering excitability
Source: Cell Death Discov. 2026 Oct 15;12:432. doi: 10.1038/s41420-026-03366-7 (PMC13720065; doi:10.1038/s41420-026-03366-7)
Supplement: Supplementary file 3 [file 41420_2026_3366_MOESM3_ESM.docx]

**Supplementary Material: Uncropped immunoblots of Sup2B**

36KD

55KD

GAPDH

CXCR2
